# It’s Not Just Conflict That Motivates Killing of Orangutans

**DOI:** 10.1371/journal.pone.0075373

**Published:** 2013-10-09

**Authors:** Jacqueline T. Davis, Kerrie Mengersen, Nicola K. Abram, Marc Ancrenaz, Jessie A. Wells, Erik Meijaard

**Affiliations:** 1 School of Mathematical Sciences, Queensland University of Technology, Brisbane, Australia; 2 Durrell Institute for Conservation and Ecology, School of Anthropology and Conservation, University of Kent, Canterbury, Kent, United Kingdom; 3 HUTAN –Kinabatangan Orang-utan Conservation Programme, Sandakan, Sabah, Malaysia; 4 Borneo Futures Project, People and Nature Consulting International, Jakarta, Indonesia; 5 ARC Centre of Excellence for Environmental Decisions, University of Queensland, Brisbane, Australia; 6 School of Archaeology & Anthropology, Australian National University, Canberra, Australia; Midwestern University & Arizona State University, United States of America

## Abstract

We investigated why orangutans are being killed in Kalimantan, Indonesia, and the role of conflict in these killings. Based on an analysis of interview data from over 5,000 respondents in over 450 villages, we also assessed the socio-ecological factors associated with conflict and non-conflict killings. Most respondents never kill orangutans. Those who reported having personally killed an orangutan primarily did so for non-conflict reasons; for example, 56% of these respondents said that the reason they had killed an orangutan was to eat it. Of the conflict-related reasons for killing, the most common reasons orangutans were killed was fear of orangutans or in self-defence. A similar pattern was evident among reports of orangutan killing by other people in the villages. Regression analyses indicated that religion and the percentage of intact forest around villages were the strongest socio-ecological predictors of whether orangutans were killed for conflict or non-conflict related reasons. Our data indicate that between 44,170 and 66,570 orangutans were killed in Kalimantan within the respondents’ active hunting lifetimes: between 12,690 and 29,024 for conflict reasons (95%CI) and between 26,361 and 41,688 for non-conflict reasons (95% CI). These findings confirm that habitat protection alone will not ensure the survival of orangutans in Indonesian Borneo, and that effective reduction of orangutan killings is urgently needed.

## Introduction

Kalimantan, Indonesian Borneo, is one of the last natural refuges of the orangutan, but it is a tenuous existence for this iconic species. Despite government laws on protection of habitat and wildlife, and cultural restrictions on hunting, the population of orangutans has declined alarmingly over the last few decades [1]–[3]. Human population pressure and agricultural expansion has led to substantial forest clearing and degradation on Borneo and Sumatra, which has in turn reduced orangutan habitat and food sources. Beyond the direct effects of forest loss on orangutan survival and reproduction, there is a well-established argument that loss of habitat and food sources has increased human-orangutan contact and conflict, leading to conflict-motivated killing by humans [3]–[5].

Conflict-motivated killing by humans - where conflict is defined as a negative interaction involving actual or feared harm, damage or interference with activities - is indeed well recognised as an issue among conservation organisations. Conservation organisations and governments have established several rescue units to assist in cases where orangutans cause conflicts with farmers and oil palm plantations in both Sumatra and Borneo. The Indonesian and Malaysian governments, the legally responsible agents for Sumatran and Bornean orangutans, are aware of the links between habitat loss, increasing human-orangutan conflicts, and declining orangutan populations, and have developed policies to counteract them (e.g., [6]), but these have so far failed to effectively reduce the occurrence of conflict. Industry groups and communities who operate, live or work close to orangutan habitat are similarly aware that orangutans can cause damage to crops [7], [8], but coherent long-term strategies have yet to be developed and implemented to prevent conflict-motivated killings of orangutans.

It is also well recognised, although substantially less publicised, that orangutan killings occur outside situations of direct conflict. Results from interview-based surveys suggest that orangutans in both Sumatra and Borneo are sometimes killed for food, medicinal purposes, or to obtain young animals for the pet trade [4], [9]. This issue was recently highlighted in a paper by Meijaard *et al.* [5]. Based on a recent survey of almost 7,000 villagers in 687 villages within the orangutan distribution range in Kalimantan, the authors reported a surprisingly high level of killing, and found that although the majority of reasons given for killing involved conflict, a substantial proportion of killings were not for this reason.

According to Meijaard *et al.* [5], among reliable respondents who had seen an orangutan around their village, 15% reported that agricultural conflicts with orangutans had occurred at some time in their village. A quarter of all sampled villages had one or more reliable respondents who reported that an orangutan had been killed in the village at some time during their residence, and in one-fifth of sampled villages at least one respondent had personally killed an orangutan. Further to this, 40% of all reliable respondents who had seen an orangutan around their village reported that an orangutan had been killed within their village at some time during their period of residence. Common reasons for orangutan killings in the village included personal consumption, self-defence, and crop raiding, though a multitude of other reasons were given by respondents.

This paper aims to provide further insight into the different reasons why orangutan killings occur. Specifically, three main questions are addressed: 1) What are the reasons for killing orangutans in Kalimantan, and what role does conflict play in these deaths?; 2) What socio-ecological factors predict reasons for killing?; and 3) How many orangutans are killed for conflict and non-conflict reasons?

## Methods

**Figure 1:**
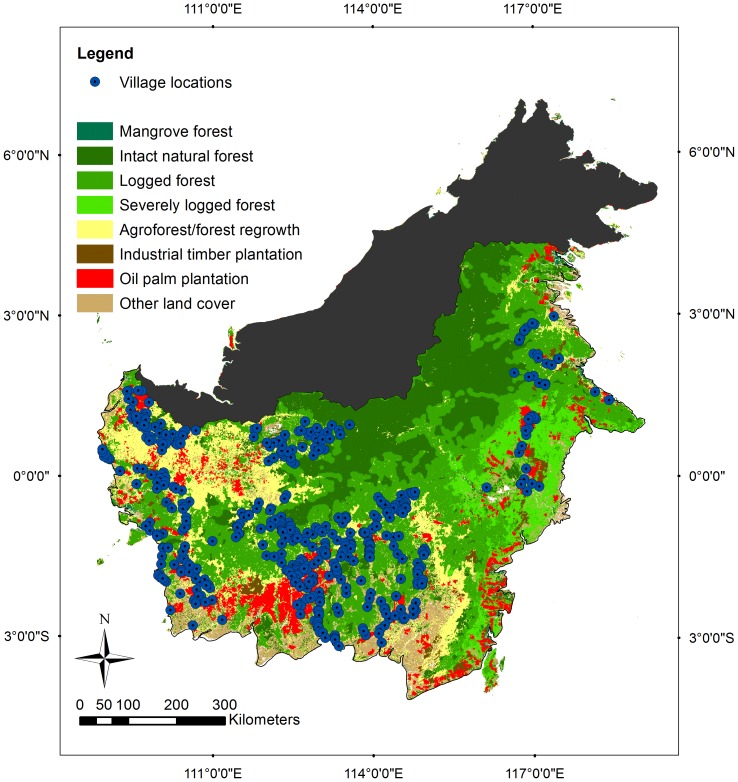
Survey locations and land cover in Kalimantan.

Meijaard *et al.* [5] provided details of the design and conduct of the survey on which the current analysis is based. In brief, the survey involved 19 conservation non-governmental organisations and was conducted between April 2008 and September 2009 in three provinces in Kalimantan, where orangutans were known to occur. A stratified random sample (across high/medium/low risks of land use change) of 40% of villages in the target region resulted in an initial selection of 687 villages, which was subsequently reduced to 476 villages (see 5). A questionnaire was delivered by interview to approximately 10 residents in each of the villages and comprised questions on basic demographics, assessment of interviewee reliability, perceptions and experiences relating to orangutans, knowledge of national and customary laws, and forest use and perceptions. See Figure 1 for a map of the survey locations.

We distinguish at all times between ‘individual level’ responses (i.e. reported killings by the respondent personally), and ‘village level’ responses (i.e. respondents’ reports of orangutan killings by others in their village). All individual level analyses were restricted to reliable respondents, i.e., those who could correctly identify an orangutan and at least one of the other nominated primate species, and those who had reported personally killing at least one orangutan (*n* = 328 orangutans killed by 143 villagers). All figures were constructed from individual level responses, and analyses of socio-ecological predictors and calculation of total numbers killed were restricted to individual level responses. We did not use village level responses for these analyses to avoid introducing bias due to over-counting at the village level, and due to potentially increased error rates introduced in respondents’ perceptions of the reasons for actions by others. Responses of ‘other’ and ‘don’t know’ were excluded from all analyses.

### Ethics Statement

Because the Nature Conservancy does not have a specific institutional review board or ethics committee, the interview survey approach was reviewed and approved by the Nature Conservancy’s social science specialist. Written approval for the interview survey was given by the Indonesian Directorate General of Forest Protection and Nature Conservation, and appropriate documentation was obtained to conduct the surveys in the Kalimantan provinces. Before beginning the survey, potential participants in the surveys were informed of the goal of the interviews and assured that the data would be analysed anonymously through a statement read by the interviewer (see Supplementary Information in [5] for details of this statement). Interviews were then conducted based on verbal consent to participate.

The current study focuses on two of the survey questions, the first regarding reasons why orangutans were killed around the village, and the second regarding reasons why the respondent personally killed an orangutan. Commensurate with the three main questions posed for this study, three stages of statistical analyses were undertaken.

### Reasons for Killing and the Role of Conflict

Reported reasons for killing were classified into 12 groups, which were further classified as conflict, non-conflict or unidentified reasons as follows: (i) Conflict reasons: pest, fear/self-defence, paid or forced to kill, orangutans interrupted logging or forestry operations; (ii) Non-conflict reasons: traditional medicine, food, to sell or keep young as pets, hobby/sport hunting, for other trade of animals or meat, and killed accidentally or opportunistically while hunting other animals, e.g., dogs or snares; and (iii) Unidentified reasons: don’t know, other. The categorisation of reasons for killing into “conflict” and “non-conflict” depended on the degree to which each reason was consistent with the narrative of orangutan killing as a result of human-orangutan conflict driven by habitat loss. “Conflict” reasons were those in which humans had killed orangutans due to actual or feared damage or harm by the orangutans, while “non-conflict” reasons included situations in which humans had gone to some effort to kill orangutans for a particular purpose, or killed by accident within a forest habitat. Examples of the types of statements included in each category are given in the Results section.

### Socio-ecological Predictors of Killings

Associations between conflict and non-conflict killings and socio-ecological variables were investigated using boosted regression trees (BRT) and Classification and Regression Trees (CART). In a CART analysis, the response variable is described by a cascading series of binary splits of the explanatory variables; this is often represented as a tree-like structure with the final nodes representing homogeneous subsets of the responses. The selection of variables, the placement of the variables in the tree model, and the choice of location of the binary split are all data-dependent and determined by the model. A Boosted Regression Tree (BRT) is a form of CART in which many shallow trees, based only on the primary splits, are formed on random subsets of the data and are then combined. The two approaches facilitate strong ecological inference, since BRT and its analogues have been shown to provide improved predictive performance [10], whereas CART models are more interpretable and can more clearly highlight complex interactions. Moreover, the robustness of CART models can be enhanced through cross-validation and controls on the complexity of the tree.

Statistical analyses to answer the second study question focused on three groups of explanatory variables: (i) individual level covariates; (ii) village level covariates; and (iii) land use covariates. The individual level covariates included gender, age, religion, ethnic group and time spent in the forest ( = FT, a measure of how much time the respondent spent in the forest each year). The village level covariates included population size, schools per individual, primary ethnic group, and religion (% Christian, % Islam, % other). The land use covariates included percentage of vegetation and land use cover (such as intact forest, logged forest, industrial timber plantation and oil palm plantation), at a range of distances around the village (i.e. within circles of radius 3, 5, 10, and 20 km). For details on these variables, see Meijaard et al. [11].

The BRT models were fitted using the R statistical software packages ‘gbm’ and ‘gbm.step’, with the following specifications: a continuous response with a Laplace (absolute deviation) or Gaussian (squared error) loss function, 5,000 trees with an interaction depth of 3 (i.e. including multi-way interactions), bagging fraction of 0.5 (i.e., 50% random samples used for fitting the trees), training fraction of 0.8 (i.e., 20% data reserved for independent model testing), and five-fold cross-validation. The performance of the model was also assessed using five-fold cross-validation and the adequacy of the choice of the number of trees was confirmed. The CART models were fitted using the package ‘rpart’, with five-fold cross-validation, construction of up to 5,000 trees, and rigorous cost-complexity criteria (cp = 0.02, min split = 20, max depth = 5). The goodness of fit of the models was evaluated using estimates of deviance and correct classification (overall, sensitivity, specificity) of predicted compared with observed responses.

### Number of Orangutans Killed for Conflict and Non-conflict Reasons

We calculated the proportions of conflict/non-conflict killings and used these to calculate estimates of the number of orangutans killed for conflict and non-conflict reasons. The following assumptions and caveats apply to this analysis: 1) Responses were only included from villagers who could reliably identify orangutans, and who answered all questions on whether they had killed an orangutan, how many orangutans they had personally killed, and reasons for killing; 2) We weighted estimates by province population of males (see [5]), but not by any other variables, e.g. age; 3) Only four women reported reasons for killing orangutans, making it impractical to reliably estimate the total number killed by women. We therefore based calculations on reports from reliable, male respondents only.

We calculated the total number of orangutans reportedly killed by sample respondents in Central Kalimantan, East Kalimantan and West Kalimantan, for (a) conflict and (b) non-conflict reasons. Calculations were conducted from individual level responses and restricted to reliable respondents who reported personally killing at least one orangutan. Totals were based on the respondents’ active hunting lifetimes to date, and so varied according to the age of the respondent (*M* = 41 years, *SD* = 12 years, range = 17 to 90 years). We then multiplied those totals by the number of reliable males in the sample for each province, for (a) conflict and (b) non-conflict, to get a total estimate per province of the number of orangutans killed for each category of reasons. Finally, we weighted province estimates by province population, to get total weighted estimates of the number killed for (a) conflict and (b) non-conflict in all of Kalimantan. The precise working for these estimates is the same as that used in [5], and calculation methods are given in the supplementary material (Appendix S1).

## Results

### Reasons for Killing and the Role of Conflict

#### Description of conflict and non-conflict reasons for killing orangutans

This section gives examples of the reasons villagers gave for killing orangutans, and how these responses were categorised first into general reasons for killing, and then into broader conflict and non-conflict categories of reasons. The sections below give details on the number of villagers reporting killings in each category, on an individual level and on a village level.

Villagers gave a variety of reasons for the killing of orangutans in their village, and also for why they had personally had killed an orangutan. The following quotes are English translations of the original responses. Orangutans were sometimes killed out of fear or in self-defence, and this reason included responses such as “*for fear of the orangutan when it was in the woods during hunting*” (60 year old Muslim male, Melayu tribe, talking about an orangutan he had personally killed). Orangutans were also killed for interfering with crops, and this reason included explanations such as “*for disturbing the durian* [*Durio* sp.] *orchard*” (38 year old Christian male, Dayak Embaloh tribe, talking about an orangutan he had personally killed). Fear, self-defence, and interfering with crops were categorised as “conflict” killings.

Another “conflict” reason for orangutans to be killed was due to conflict with the operations of a company; villagers gave explanations such as “*killed by the company*” (45 year old Christian male, Dayak Ngaju tribe, talking about an orangutan killed in his village). Orangutans were also killed during conflict related to deforestation or for interfering with forestry, with explanations such as “*clearing of forests for oil palm*” (35 year old Muslim male immigrant from Java, talking about an orangutan killed in his village), and “*only killed for interrupting timber operations*” (36 year old Christian male, Dayak Selakau tribe, talking about an orangutan he had personally killed).

Villagers also identified a number of reasons for killing orangutans that were designated “non-conflict” killings. A common non-conflict reason for which orangutans were killed was for food, with explanations such as “*just to eat*” (25 year old Muslim male, Dayak Ngaju tribe, talking about an orangutan killed in his village), “*meat is consumed*” (42 year old Christian male, Dayak Kantuk tribe, talking about an orangutan killed in his village), “*sold for people to eat*” (26 year old Muslim female, Dayak Ngaju tribe, talking about an orangutan killed in her village), and “*if you happen to see an orangutan it is shot to sell the meat to Dayaks*” (30 year old Muslim male, Melayu tribe, talking about an orangutan killed in his village).

Orangutans were sometimes killed accidentally or opportunistically during hunting of other animals. Villagers gave explanations such as “*by chance happened to be killed while boar hunting*” (27 year old Christian male, Dayak Ngaju tribe, talking about an orangutan killed in his village), and “*while hunting pigs I met an orangutan*” (68 year old Christian male, Dayak Kapuas Ngaju tribe, talking about an orangutan he had personally killed). These accidental killings were included in the non-conflict category of responses.

Orangutans were also killed for traditional medicine, with villagers giving explanations like “*for sale of gall bladder*” (32 year old Christian male, Dayak Jelai tribe, talking about an orangutan killed in his village). Orangutans were sometimes killed to obtain young orangutans to trade, with explanations such as “*to take the young*” (30 year old Muslim male from Java, talking about an orangutan killed in his village), and “*to sell their young*” (52 year old Christian male, Dayak Ngaju tribe, talking about an orangutan he had personally killed). Some orangutans were killed for sport, with explanations such as “*try it for fun*” (70 year old Muslim male, Kutai tribe, talking about an orangutan he had personally killed). These reasons were included in the non-conflict category of responses.

#### Frequency of reasons for killing: Respondent level

**Figure 2:**
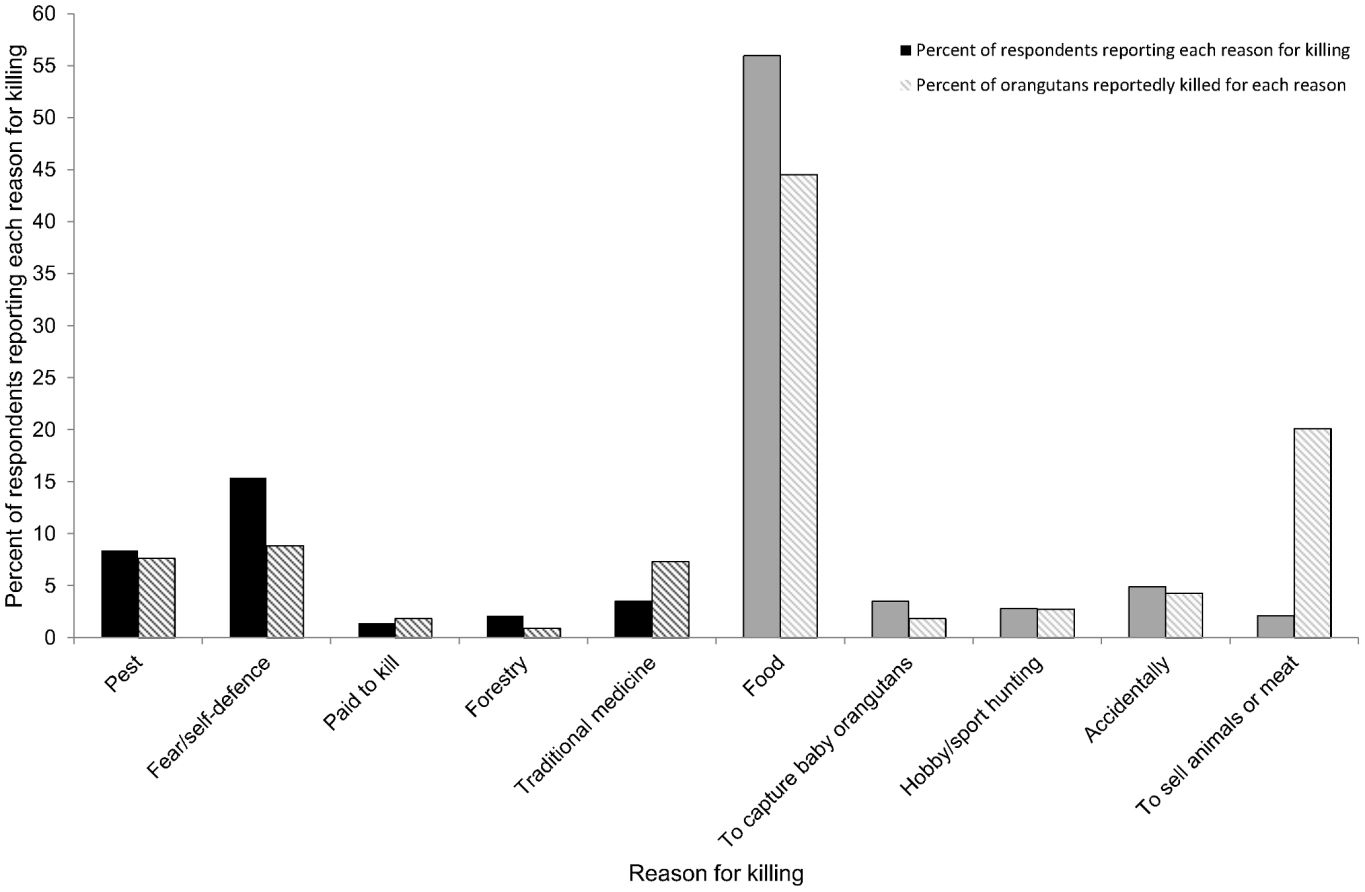
Frequencies of reasons for killing orangutans. Black columns correspond to conflict reasons; grey columns correspond to non-conflict reasons.

Respondents were unlikely to report having personally killed an orangutan and of the 4,839 villagers who provided an answer to this question, 97% declared that they personally had never killed an orangutan. Further, even among respondents who identified a reason for killing orangutans, not all could reliably identify an orangutan on sight (see Figure S1 for a flow diagram of the attrition of sample size). In total, 143 reliable respondents (2.96% of the total original sample) said that they had personally killed at least one orangutan and gave a reason (excluding “*I don’t know*”, or nonsensical responses) for this killing (see supplementary Table S1 for frequencies of orangutans killed and villagers reporting reasons for killing). See Figure 2 for a display of the percentage of villagers who reported killing orangutans for conflict and non-conflict reasons, and the percentage of orangutans reportedly killed for each reason. Of reliable respondents who gave a reason for personally killing an orangutan, 27% gave a conflict-related reason, with fear or self-defence being the most common (15% of responses, *n* = 29 orangutans from 22 villagers). The second most likely conflict reason for killing was because the orangutan was being a pest by crop-raiding or interfering with crops (8% of responses, *n* = 25 orangutans from 12 villagers). Less common conflict-related reasons for killing were for pay (1% of respondents, *n* = 6 orangutans from 2 villagers) and because the orangutan interrupted forestry operations (2% of respondents, *n* = 3 orangutans from 3 villagers).

Non-conflict reasons for killing orangutans made up 73% of valid responses from respondents who reported personally killing an orangutan. Killing an orangutan to eat it was the most commonly reported response, with 56% of respondents identifying food as the primary reason they personally had killed an orangutan (*n* = 146 orangutans from 80 villagers). Other non-conflict reasons for killing orangutans were less common, and included accidental killings whilst hunting other animals (5% of respondents, *n* = 14 orangutans from 7 villagers), to capture baby orangutans (3% of respondents, *n* = 6 orangutans from 5 villagers), for traditional medicine (3% of respondents, *n* = 24 orangutans from 5 villagers), for hobby or sport hunting (3% of respondents, *n* = 9 orangutans from 4 villagers), and for other trade of animals or meat (2% of respondents, *n* = 66 orangutans from 3 villagers). These percentages, along with the regression trees and calculations of total numbers of orangutans killed, do not include one outlier: a reliable respondent who reported having killed 100 orangutans for traditional medicine.

#### Frequency of reasons for killing: Village level

Reasons for killing at the village level closely reflected what was reported at the individual level. In 26% of the 476 villages surveyed, at least one of the villagers surveyed reported that an orangutan had been killed in the last year in the vicinity of the village. Overall, 12% of the villages had at least one account of a conflict-driven killing, while 17% of the villages had one or more reports that an orangutan had been killed for non-conflict related reasons. Of the conflict related killings, 12% of the villages had accounts of orangutans being killed through fear or self-defence; and, 11% and 2% of the reported killings were due to orangutans interfering with crops or forestry operations respectively. Lastly, in 1% of the villages, at least one villager reported that an orangutan had been killed there for pay. Of the non-conflict reasons for orangutan killings, 30% of the villages had at least one report that orangutans had been killed for food. Other non-conflict reasons included: to capture the baby orangutans (4% of the villages), for traditional medicine (2%), for hobby or sport hunting (2%), accidentally while hunting other animals (1%), and to trade the animals (1%).

### Socio-ecological Indicators of Killings

#### All reasons for killing

**Figure 3:**
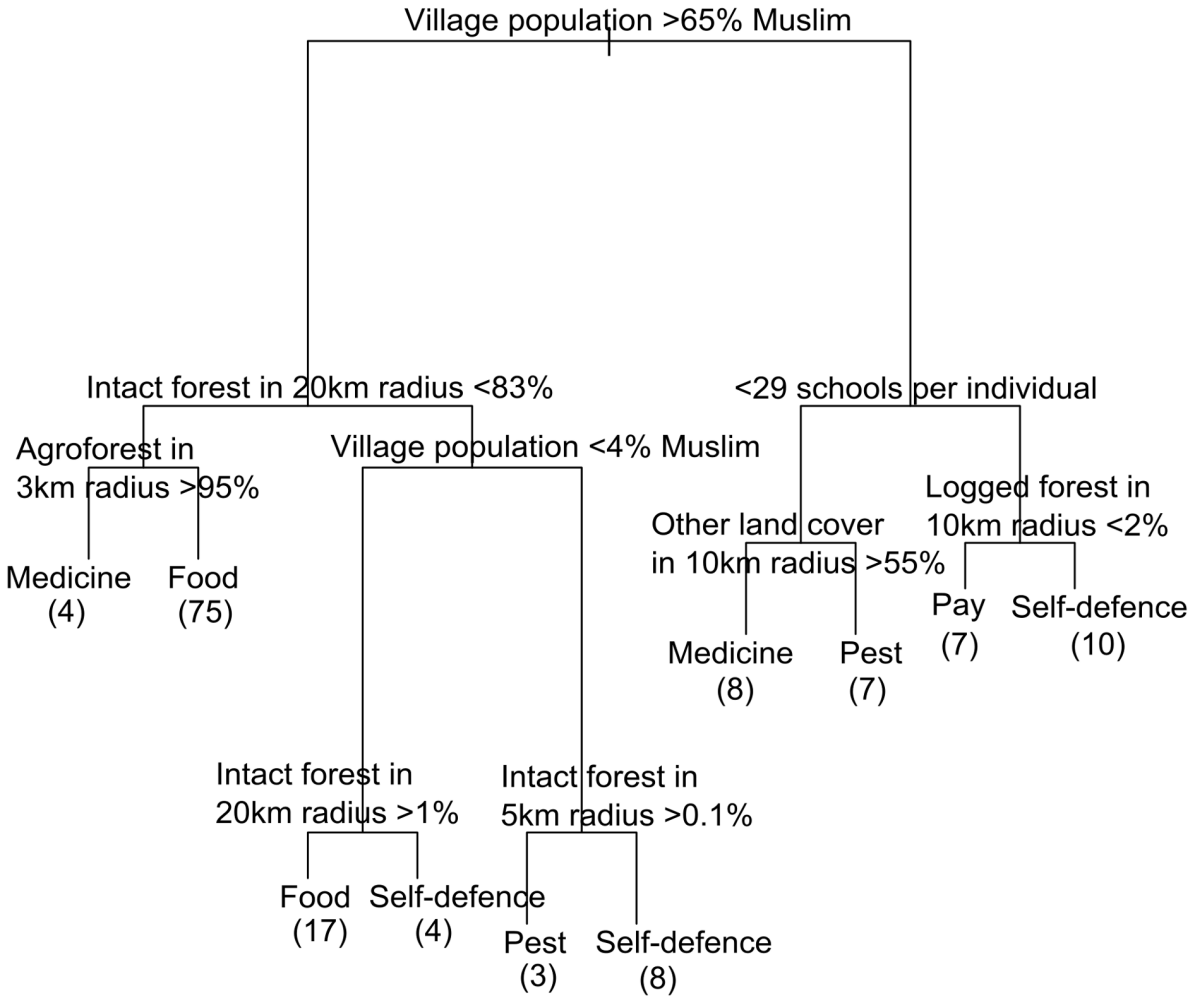
Predictive model for reasons for killing orangutans, based on CART analyses. The “yes” condition is on the left. For example, if the village population is greater than 65% Islam, and the amount of intact forest within a 20 km radius of the village is less than 83%, and the amount of agroforest in a 3 km radius of the village is more than 95%, villagers are likely to have killed an orangutan for use in traditional medicine. However, if the amount of agroforest in a 3 km radius is less than 95%, villagers are likely to have killed an orangutan to eat it. The number in brackets denotes the number of respondents that fall into each final category. For example, 75 villagers had killed an orangutan for food, and had less than 95% agroforest in a 3 km radius, and had less than 83% intact forest in a 20 km radius, and lived in a village where more than 65% of the population are Muslim.

Reasons for killing were described by a complex interplay of social and ecological variables. Based on the CART model (see Figure 3), the primary indicators were religious composition of the village, land cover variables including the amount of intact forest, agroforest, and logged forest around the village, and the number of schools per individual (an indicator of socioeconomic status). The model selected two groups of villagers most likely to have killed an orangutan for food: first, those in villages without a high proportion of surrounding agroforest, with some intact forest around the village and with a medium-high Muslim population; and second, those in villages surrounded by a lot of intact forest and with a low Muslim population. Three groups were identified that were most likely to have killed an orangutan in self-defence: first, those in villages surrounded by some logged forest, with many schools per individual (relatively high socioeconomic status), and a medium-low Muslim population; second, those in villages surrounded by no intact forest, with some Muslim population; and third, those in villages surrounded by a large amount of intact forest, with a low Muslim population. Other primary groups identified by the model were likely to have killed an orangutan for medicine, pay, or as a pest.

#### Predictors of conflict

Overall, the model correctly classified 73% of killings into conflict or non-conflict related categories. Whether a killing occurred for a conflict or non-conflict reason depended on a complex combination of social and ecological variables. Based on the BRT analyses, the dominant indicators of conflict were the religious composition of the village, and whether the village received income from growing vegetables. Further important variables were the percentage of logged forest within a 10 km radius of the village, and the age of the respondent.

**Figure 4:**
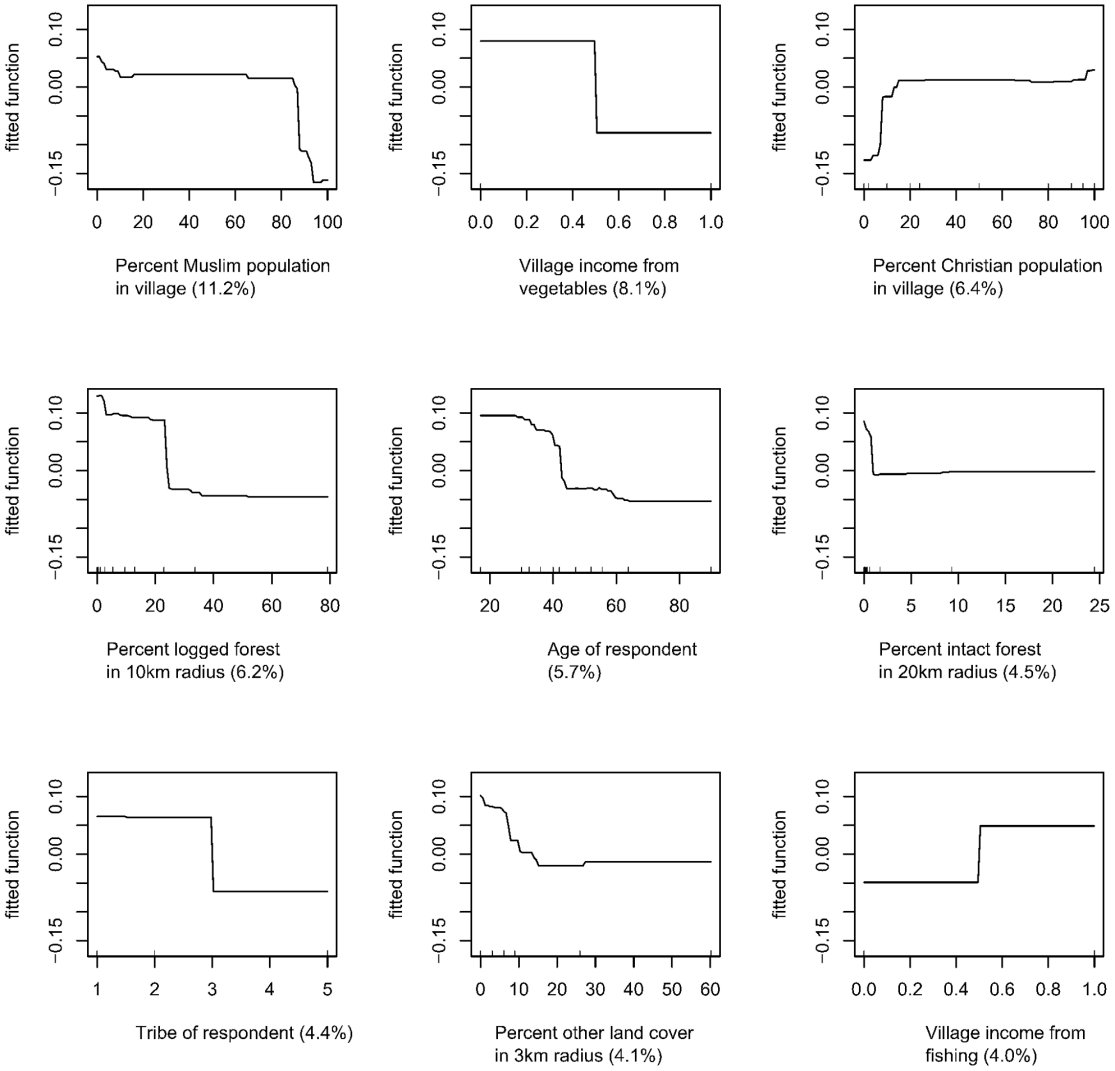
Marginal effect of the top nine predictors of non-conflict killing based on BRT analyses. Vertical axis is log-odds of a non-conflict killing, and the horizontal axis is units of the predictor as identified below each line graph. The number in parentheses is the relative importance of the variable in the BRT model, and the line describes the shape of the bivariate relationship between the predictor and the log-odds of a non-conflict killing. For example, the most important predictor of a non-conflict (compared to a conflict) killing is the percentage of the village population who are Muslim. The shape of the line shows that the log odds of killing an orangutan for a non-conflict reason stays constant as the percentage of Muslim villagers increases, until the village is about 80% Muslim, where the log odds of a non-conflict killing decrease sharply.

As seen in Figure 4, the analysis revealed strongly nonlinear effects of the predictors on the risk (expressed as log-odds) that a killing was for a conflict-related purpose, such that villages with a high proportion of Muslim residents were much more likely to report killing an orangutan for a conflict reason. Similarly, the odds of non-conflict killing increased then plateaued with a larger percentage of Christians in the village, but decreased then plateaued with a larger percentage of logged forest in a 10 km radius around the village. Older respondents were more likely to have killed orangutans for conflict reasons than were younger respondents, and respondents were more likely to have killed an orangutan for non-conflict reasons if they were of Dayak ethnicity (used here as a loose term for over 200 riverine and hill-dwelling ethnic subgroups, located principally in the interior of Borneo, see [12]).

The CART analysis confirmed the interacting nature of the variables in predicting conflict and non-conflict killing (Figure S2). The dominant variables were primarily village-level variables, including the religious composition of the village, and the amount of agroforest and oil palm plantation surrounding the village. The only important individual-level variable identified in the CART analysis was respondent age, which interacted with village-level variables in a number of complex ways to determine whether a killing was conflict or non-conflict related. Orangutans were most likely to have been killed for a non-conflict reason in villages without a high Muslim population, and surrounded by some agroforest, and by younger respondents. The second largest grouping of non-conflict killings was in villages without a high Muslim population, surrounded by some agroforest, and where the village did not source any income from vegetables.

#### Hunting for food

**Figure 5:**
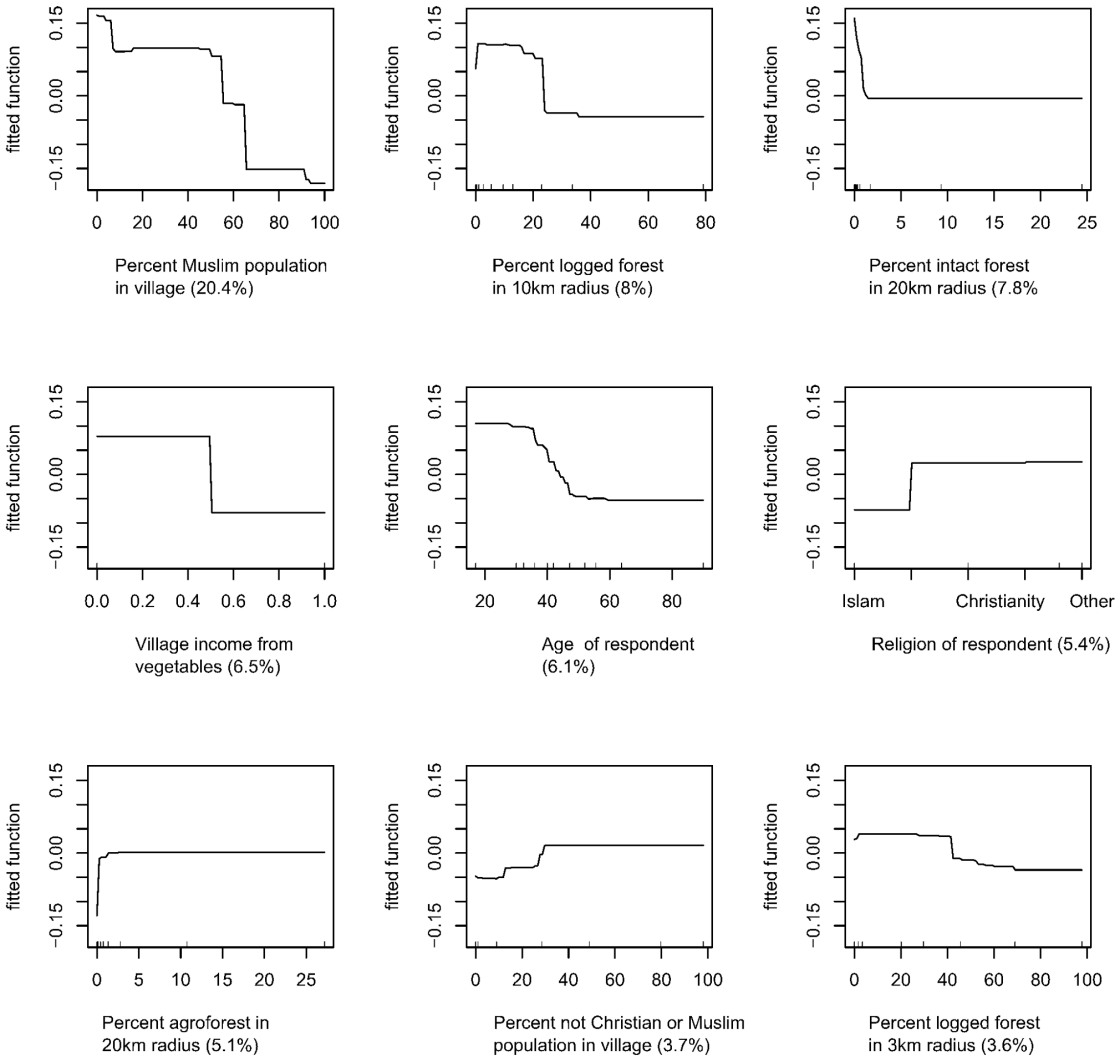
Marginal effect of the top nine predictors of food-related killing based on BRT analyses. Vertical axis is log-odds of a food-related killing and the horizontal axis is units of the predictor as identified below each line graph. The number in parentheses is the relative importance of the variable in the BRT model.

Since consumption of orangutan meat accounted for most of the non-conflict related killings (80/104 respondents and 146/265 orangutans) BRT and CART analyses were undertaken to identify the main predictors of hunting for food. According to the BRT results, the dominant explanatory variables were the religious composition of the village and the amount of logged and intact forest surrounding the village, followed by village income from vegetables and the respondent’s age and religion. The marginal relationships of these variables on the log-odds of a food-related killing are shown in Figure 5. The BRT analysis revealed that the risk of a food-related killing was highest for individuals who were not Muslim, and were younger; and in villages that had more agro-forest/forest re-growth, less intact forest, and less logged forest around the village. The model fit well, with overall 60% correct classification.

The results of the CART analysis confirmed the BRT analysis, as shown in Figure S3. A complex set of inter-related variables predicted whether orangutans were killed for food. The religious composition of the village was important to determining whether orangutans were killed for food, as was the amount of logged and agroforest around the village. Individual villagers’ age and religion were also important determinants of whether they had killed an orangutan for food or for some other reason. Orangutans were most likely to have been killed for food in villages with a low proportion of Muslim population, a high amount of logged forest around the village, and no village income from vegetables.

### Number of Orangutans Killed for Conflict and Non-conflict Reasons in Kalimantan

We estimated that between 16,275 and 35,203 orangutans were killed in Kalimantan for conflict reasons, within the respondents’ active hunting lifetimes (95% CI lower = 16,275, estimated total = 25,739, 95% CI upper = 35,203). We estimated that between 23,221 and 37,279 orangutans were killed in Kalimantan for non-conflict reasons, within the respondents’ lifetimes (95% CI lower = 23,221, estimated total = 30,251, 95% CI upper = 37,279).

## Discussion

### Methodological Considerations

Various potential sources of biases exist for surveys like the one reported in this paper, including respondent and social desirability biases. First, surveys can be biased if they do not sample the correct population. Our survey was designed to sample the population of villagers who knew about orangutans. We used several methods to ensure that we did in fact sample this population. We used a stratified random sampling method to sample villages in particular geographic areas, and areas which were high-risk for orangutans. Within villages, we asked interviewers to identify respondents who knew about orangutans, as the most reliable sources within the village. We also tested whether or not respondents could reliably identify an orangutan, using a picture-based knowledge validation method. Those respondents who could not reliably identify an orangutan were excluded from all analyses. Such step-wise methods to ensure and enhance data integrity mean that we are reasonably satisfied that the data represent an adequate sample of the population of interest.

Second, survey results can be biased if respondents refuse to answer particular questions within the survey. These are usually sensitive questions, for which the respondent may feel embarrassed answering, or fear reprisal from third parties for an honest answer [13]. We quantified the possible impact of this non-response bias on our results by calculating what percentage of respondents identified killing an orangutan but refused to provide a reason why they had killed it. Overall, 14% of respondents who identified killing an orangutan did not provide a reason for killing it. However, the probability of respondents not answering the question was not related to any demographic variables, including the respondent’s ethnic group (χ^2^(3, *N* = 174) = .293, *p* = .293), religion (χ^2^(2, *N* = 174) = 4.52, *p* = .104), or the province in which the data were gathered (χ^2^(2, *N* = 174) = 7.36, *p* = .061). These results suggest that the missing responses were distributed randomly among respondents, and therefore that it is unlikely that the respondents who did not give a reason for killing would have given substantially different reasons to the respondents who did give a reason for killing. Our overall estimates should therefore be unaffected by the non-responses.

Third, lying, self-deception, and presenting false information to make oneself look better, are all strategies that respondents in any survey may use if they feel they will be held accountable for their answers, either by the interviewer or by someone else [14]. The presence of a face-to-face interviewer may in theory exacerbate respondents’ tendency to lie or exaggerate ([15], cf. [16]). However, decades of research on social desirability bias has concluded that this effect results in *under*-reporting of illegal activities [13], such as hunting legally protected orangutans. It is reasonable to expect that villagers may have adjusted their responses based on their expectations of the interviewer’s values, and on the possibility of reprisals from authorities. However, these adjustments, if they exist, in all probability mean that our results underestimate the number and proportions of orangutans killed for non-conflict reasons.

Finally, human-orangutan interaction is a complex issue that necessitates some simplification to enable quantitative analysis. Reasons for killing orangutans are affected by a complex interplay of social, economic and historical factors, which cannot all be fully described by a single reason for killing. Complicating the issue is the fact that 39% of villagers killed more than one orangutan, potentially for different reasons (see supplementary Table S3 for frequencies of the number of orangutans killed vs. the number of reasons given). In fact, villagers could have killed several orangutans for one reason, one orangutan for several reasons, or several orangutans for several reasons. We chose to assign one reason to all of the orangutans killed by each individual because this closely reflects what villagers actually reported. Despite having the opportunity to provide more than one reason for killing orangutans, the vast majority of villagers chose to provide only a single reason (only 7% of villagers provided a second reason, and only 2% provided a third reason). Substituting the second or third reasons given by individuals did not change the makeup of each category by more than 2%, did not change the composition of the overall conflict and non-conflict categories by more than about 1% (for exact numbers, see Table S2), and did not change which were the most commonly reported reasons for killing. No relationship was apparent between the number of orangutans killed and the number of reasons given–it was not the case that villagers who killed more orangutans gave more reasons. The approach presented in this paper was selected as the most robust possible, given the difficulties inherent in any attempt to quantify such a complex phenomenon.

### Drivers of Orangutan Conflict and Killing

Although conflict is a major driver for killing orangutans, a substantial proportion of killings occur for non-conflict reasons, with food being the principal reason for orangutan killing overall. In fact, more orangutans are killed for food than for any other reason. The second most common reason for which orangutans are killed is out of fear or self-defence. Additionally, some orangutans are killed for encroaching on crops or property as pests. According to villagers’ reports, however, the number of orangutans killed for pest control is extremely small in comparison to the number killed for food.

Certain social and ecological factors were important in predicting the reason for orangutans being killed, and whether this reason was conflict or non-conflict related. The most important indicator of conflict or non-conflict killing of orangutans was religion, as demonstrated by several of our analyses. Villagers who lived in an area with a high proportion of Muslims, or who were Muslim themselves, were less likely than non-Muslims to kill orangutans for non-conflict reasons, and specifically for food.

Also important was the land cover composition of the surrounding landscape. Orangutans were more likely to be killed for food in villages surrounded by some intact forest. This may be due in part to villagers being more likely to encounter orangutans in such a setting; however, these encounters were still more likely to result in killing for a non-conflict reason, and particularly for food, than for a conflict reason.

Village income, particularly from vegetables, was selected as an important predictor of killing for non-conflict reasons and for food. Specifically, individuals in villages with low income from vegetables were more likely to have killed an orangutan for food (given the context of religious and land cover variables as previously discussed). One possible explanation for this result is a situation in which villagers use orangutans as an alternative food or income source in the absence of local farming for cash income.

The number of orangutans estimated to have been killed within the survey respondents’ active hunting lifetimes gives cause for alarm. Current estimates put the population of orangutans in Kalimantan somewhere between 38,330 and 40,000 in total, and our lowest estimate of the number killed in living memory is 44,165. One reason for our estimate being distressingly large in relation to this estimate of the total population of orangutans, may be that the population figures above are an underestimate of the true population, a possibility noted in the estimate’s original report [17]. Forthcoming estimates based on new survey data are expected to revise this population estimate upwards. Another, more unsettling, reason may be that the population of wild orangutans has in fact halved in the respondents’ lifetimes, due to hunting and conflict with humans. We do not have the information to suggest which explanation is more likely.

We present all of our results with the caveat that assigning a single reason for any individual’s actions is contentious, given that all actions take place in a larger social, economic and historical context. It is difficult to determine an exact rationale for an individual’s behaviour and create mutually exclusive categories of reasons for killing, and of conflict and non-conflict. For example, an individual who states that he has killed an orangutan for food (non-conflict reason), cannot necessarily be determined to be free from the undercurrent of historical, economic and social repercussions of logging and habitat loss (conflict reasons). We present a simplification of these reasons, with the aim of identifying the primary, proximal drivers for orangutan killings, and using these quantitative methods to explore their socio-ecological correlates, and thereby clarify some of the complexity surrounding this issue.

### An Uncomfortable Truth About Orangutan Decline

Our findings may make unpleasant reading for many. Initial reports of hunting take-off levels of orangutans [5] were greeted by disbelief and protest. A spokesman for the Indonesian forestry ministry described the report’s findings as "*bombastic*" and said he doubted they were true [18]. Dayak representatives protested saying that such killings were nearly non-existent and would only occur when people were threatened by orangutans [19]. Many Indonesian conservation scientists, including some who had helped coordinate the interview surveys, have expressed their doubt about the veracity of the findings and conclusions, as expressed in personal communications to author EM. The consensus among many of these people appears to be that habitat loss is a far bigger driver of orangutan decline than either conflict or non-conflict killings. We explore why there appears to be a discrepancy about how outside observers perceive the severity of the orangutan killing problem, and how this is reported by the interview respondents.

First, orangutans are rare. With densities rarely exceeding two animals per km^2^, wild orangutans are scarce, and few people will ever encounter one. Compared to other animals that are hunted in villages in Borneo, the occasional killing of an orangutan might appear insignificant. The number of pigs, deer, muntjaks (*Muntiacus* spp.) and monkeys that are killed in Bornean villages may outnumber the number of orangutans killed by 3 orders of magnitude ([20], [21], [22], EM, unpubl. Data). Meijaard et al. [5] estimated that between 750 and 1,800 orangutans had been killed in Kalimantan in the year prior to the survey and that between 1,950 and 3,100 orangutans had been killed annually over the life time of interview respondents. This indicates an approximate killing rate of about 1 orangutan every 1.3 to 5 years per village, for the approximately 4,000 villages within the orangutan’s range in Kalimantan. These numbers might seem negligible, but they likely exceed the mortality rates that viable populations of this slow-breeding species can sustain [23].

Second, many conservation advocates may retain an idealised view of those that live in close association with tropical rainforests; a view that does not align well with reports of villagers hunting and killing orangutans for food and profit, especially when vast and complex forces such as industry growth and habitat loss can be blamed instead [24], [25]. Such views ignore substantial evidence demonstrating that orangutans have been hunted on Borneo for tens of thousands of years [26], [27], and that hunting is among the most likely reasons for the orangutan’s decline in prehistoric [28] as well as more recent times [29]. Our findings do not diminish the need to address habitat loss as a key threat to the survival of orangutans and other wildlife, but they do point to another avenue for conservation efforts; one that may be currently obscured by individual preconceived values and perceptions.

To put our findings into perspective, we offer a global comparison. Primates are hunted for food in every geographic location where they coexist with humans. The trade in primate meat has been identified as a problem for conservation efforts in Africa [30]–[33], Asia [34], [35], and Central and South America [36]–[39]. In the 2012–2014 *Primates in Peril* report, hunting was identified as a threat to survival for 19 of the top 25 most endangered primates, with 10 primate species explicitly being hunted for meat, including Eastern Lowland Gorilla (*Gorilla berengei graueri*) [40]. Hunting for meat, not habitat loss, has been identified as the greatest immediate threat to primate conservation in African forests [41], [42]. More than 20 years ago, a worldwide review of primate hunting concluded that the primary reason people in all countries kill primates was to eat them [43], and a more recent review concluded that in most cases, hunting was responsible for primate population declines well before deforestation occurred [44]. In this global context, it would in fact have been surprising if orangutans were not hunted for food.

### Constructive Ways Forward

We do not intend to accuse, or use our finding to generalise about, any particular group of people. We are aware of the heterogeneity of human perceptions and actions even within religious or ethnic groups [11]. However, we do wish to identify patterns and trends inherent within our dataset in relation to orangutan killings, and to bring to light the realistic scenarios that threaten orangutan persistence. It is vital that we understand such complex threats so that targeted conservation strategies can be developed and implemented.

The reasons orangutans are killed can be predicted with reasonable accuracy by village-level characteristics, and this may be helpful in the design of efforts for orangutan conservation. For example, the Indonesian Action Plan calls for all wild populations to be stable by 2017 [6]. For populations to be stable they have to be viable and thus have a minimum viable population (MVP) size. An understanding of this MVP size and predicted local killing rates would inform conservation managers about the most appropriate local action (e.g., increasing population size by reintroducing animals or reconnecting distinct populations through reforestation, or reducing local killing pressure). If the reduction of local killing is a focus for conservation action, an understanding of the association with social, economic and environmental factors helps in designing optimal strategies. Depending on the main reasons for killing in a particular area, such strategies could be focused on education, economic development, awareness raising, compensatory payments for conflicts, or other tools. Considering what works best in which situation should enable much more effective and efficient orangutan conservation.

To a large extent, the safeguarding of remaining wild orangutans populations on Sumatra and Borneo depends on the development and implementation of more appropriate land use policies that stop the destruction and degradation of orangutan habitats, and give people longer and more secure tenure of land, forest, and other natural resources [45]. In addition, there has to be an understanding that if current rates of orangutan killings continue, most populations will go extinct within one human generation. In this scenario, only those few populations that are well-protected or that occur in areas where killings are rare due to cultural and traditional reasons in the local community (e.g., Kinabatangan or other populations located in eastern Sabah [46], [47]) stand a chance of long-term survival. Local, forest-based communities play a crucial role in the long-term maintenance of Borneo’s forests and their wildlife. Borneo’s people still depend to a large degree on forest resources, for their livelihoods, health and spiritual well-being [11]. Respecting and formalising people’s rights to forest use would be a first step towards stabilising Borneo’s rapidly disappearing forest frontiers, while laying a basis for two-way discourse about how the challenge of orangutan killing could best be addressed. Recognising that a problem exists is only the start.

## Supporting Information

Figure S1 **Flow diagram of survey sample size.**
(TIFF)

Figure S2 **CART prediction of non-conflict (NC) versus conflict (C) reasons for killing orangutans.**
(TIFF)

Figure S3 **CART prediction of food-related (1) versus non-food related (0) reasons for killing orangutans.**
(TIFF)

Table S1 **Number of villagers reporting each reason for killing and number of orangutans reportedly killed for each reason, respondent level only.**
(PDF)

Table S2 **Comparison of primary, secondary, and tertiary reasons for killing orangutans, if more than one reason given, respondent level only.**
(PDF)

Table S3 **Number of orangutans killed vs. number of reasons for killing given, respondent level only.**
(PDF)

Appendix S1 **Calculation sheet for number of orangutans killed for each reason in respondents’ lifetimes.**
(XLS)
