# Supplementary material for: It’s Not Just Conflict That Motivates Killing of Orangutans
Source: PLoS One. 2013 Oct 9;8(10):e75373. doi: 10.1371/journal.pone.0075373 (PMC3793980; doi:10.1371/journal.pone.0075373)
Supplement: Table S1 — Number of villagers reporting each reason for killing and number of orangutans reportedly killed for each reason, respondent level only. (PDF) [file pone.0075373.s004.pdf]

	Primary reason for killing	Percent of villagers	Number of villagers	Percent of orangutans	Number of orangutans
Conflict reasons	Pest	8.4	12	7.6	25
	Fear/self-defence	15.4	22	8.8	29
	Paid to kill	1.4	2	1.8	6
	Forestry	2.1	3	0.9	3
	**Total conflict**	**27.3**	**39**	**19.2**	**63**
Non-conflict reasons	Traditional medicine	3.5	5	7.3	24
	Food	55.9	80	44.5	146
	To capture baby orangutans	3.5	5	1.8	6
	Hobby/sport hunting	2.8	4	2.7	9
	Accidentally	4.9	7	4.3	14
	To sell animals or meat	2.1	3	20.1	66
	**Total non-conflict**	**72.7**	**104**	**80.8**	**265**
	**Overall**	**100**	**143**	**100**	**328**
