# Supplementary material for: It’s Not Just Conflict That Motivates Killing of Orangutans
Source: PLoS One. 2013 Oct 9;8(10):e75373. doi: 10.1371/journal.pone.0075373 (PMC3793980; doi:10.1371/journal.pone.0075373)
Supplement: Table S2 — Comparison of primary, secondary, and tertiary reasons for killing orangutans, if more than one reason given, respondent level only. (PDF) [file pone.0075373.s005.pdf]

	Reason for killing	Percent of villagers giving this reason if only including primary reasons, substituting secondary reasons, or substituting tertiary reasons.		
		Primary	Secondary *(difference)*	Tertiary *(difference)*
Conflict reasons	Pest	8.39	10.49	8.39
			(2.10)	*(0.00)*
	Fear/self-defence	15.38	16.08	15.38
			(0.70)	*(0.00)*
	Paid to kill	1.40	1.40	1.40
			(0.00)	*(0.00)*
	Forestry	2.10	0.70	2.10
			(-1.40)	*(0.00)*
	**Total conflict**	**27.27**	**28.67**	**27.27**
			(1.40)	***(0.00)***
Non-conflict reasons	Traditional medicine	3.50	1.40	2.10
			(-2.10)	*(-1.40)*
	Food	55.94	58.04	56.64
			(2.10)	*(0.70)*
	To capture baby orangutans	3.50	4.90	3.50
			(1.40)	*(0.00)*
	Hobby/sport hunting	2.80	2.80	4.20
			(0.00)	*(1.40)*
	Accidentally	4.90	2.80	4.20
			(-2.10)	*(-0.70)*
	To sell animals or meat	2.11	1.40	2.10
			(-0.71)	*(-0.01)*
	**Total non-conflict**	**72.74**	**71.33**	**72.74**
			(-1.41)	***(0.00)***
