# Supplementary material for: It’s Not Just Conflict That Motivates Killing of Orangutans
Source: PLoS One. 2013 Oct 9;8(10):e75373. doi: 10.1371/journal.pone.0075373 (PMC3793980; doi:10.1371/journal.pone.0075373)
Supplement: Table S3 — Number of orangutans killed vs. number of reasons for killing given, respondent level only. (PDF) [file pone.0075373.s006.pdf]

	Number of orangutans killed						
	1	2	3	4	5-10	11+	**Total**
1 reason	81	27	16		5	3	**132**
2 reasons	4	1		1	1	1	**8**
3 reasons	2		1				**3**
**Total**	**87**	**28**	**17**	**1**	**6**	**4**	**143**
